# Eigenmode operation of piezoelectric resonant gyroscopes

**DOI:** 10.1038/s41378-020-00204-3

**Published:** 2020-11-30

**Authors:** Mojtaba Hodjat-Shamami, Farrokh Ayazi

**Affiliations:** School of Electrical and Computer Engineering, Georgia Institute of Technology, Atlanta, GA 30308 USA

**Keywords:** Electrical and electronic engineering, Sensors

## Abstract

The theory of eigenmode operation of Coriolis vibratory gyroscopes and its implementation on a thin-film piezoelectric gyroscope is presented. It is shown analytically that the modal alignment of resonant gyroscopes can be achieved by applying a rotation transformation to the actuation and sensing directions regardless of the transduction mechanism. This technique is especially suitable for mode matching of piezoelectric gyroscopes, obviating the need for narrow capacitive gaps or DC polarization voltages. It can also be applied for mode matching of devices that require sophisticated electrode arrangements for modal alignment, such as electrostatic pitch and roll gyroscopes with slanted electrodes utilized for out-of-plane quadrature cancellation. Gyroscopic operation of a 3.15 MHz AlN-on-Si annulus resonator that utilizes a pair of high-*Q* degenerate in-plane vibration modes is demonstrated. Modal alignment of the piezoelectric gyroscope is accomplished through virtual alignment of the excitation and readout electrodes to the natural direction of vibration mode shapes in the presence of fabrication nonidealities. Controlled displacement feedback of the gyroscope drive signal is implemented to achieve frequency matching of the two gyroscopic modes. The piezoelectric gyroscope shows a mode-matched operation bandwidth of ~250 Hz, which is one of the largest open-loop bandwidth values reported for a mode-matched MEMS gyroscope, a small motional resistance of ~1300 Ω owing to efficient piezoelectric transduction, and a scale factor of 1.57 nA/°/s for operation at atmospheric pressure, which greatly relaxes packaging requirements. Eigenmode operation results in an ~35 dB reduction in the quadrature error at the resonance frequency. The measured angle random walk of the device is 0.86°/√h with a bias instability of 125°/h limited by the excess noise of the discrete electronics.

## A new spin on microscale gyroscope operation

An alternative design and operation strategy for resonant gyroscopes delivers faster response while also achieving low levels of noise. These tiny devices measure an object’s rate of rotation, and are commonly used in consumer electronics as well as industrial and automotive applications. Resonant gyroscopes are currently limited by a tradeoff between bandwidth and signal-to-noise ratio, but Mojtaba Hodjat-Shamami and Farrokh Ayazi at the Georgia Institute of Technology have devised a solution to this problem. They employed an eigenmode operation strategy with a thin film piezoelectric-on-silicon resonant gyroscope, and demonstrated that they could routinely achieve mode-matched bandwidths of a few hundred Hertz, with greatly reduced error. This approach could greatly bolster the utility of such piezoelectric devices, but the authors note that a similar eigenmode operation strategy could also broadly optimize performance for other resonant gyroscope designs as well.

## Introduction

Resonant Microelectromechanical System (MEMS) gyroscopes have seen tremendous improvements in their performance over the past decade, enabling their adoption in various consumer, industrial, and automotive applications^1–6^. The current efforts in improving the performance of high-precision resonant gyroscopes are mainly focused on two seemingly contradictory metrics, larger bandwidths, and lower noise levels, to push the technology towards navigation applications. Considering the advantages brought about by *Q*-amplification in micromechanical resonators, mode matching has been the primary means of achieving a very high signal-to-noise ratio in Coriolis vibratory gyroscopes. Although mode matching significantly improves the noise performance, it imposes a heavy constraint on the operational bandwidth of high-*Q* devices^7–12^. Capacitive bulk acoustic wave (BAW) gyroscopes circumvent this problem by increasing the resonance frequency of the device using stiff bulk vibration modes of the structure at the cost of intricate fabrication processing to realize high-aspect-ratio gaps^13–16^. Bulk-mode resonant devices in the megahertz frequency range have also been proven to offer superior shock and vibration immunity^17–20^.

The key enabling factor for the realization of low-noise, high-bandwidth resonant gyroscopes is the utilization of a strong electromechanical transducer. Thin-film piezoelectric-on-silicon technology provides a very efficient transduction mechanism suitable for implementation of high-frequency gyroscopes without the need for submicron capacitive gaps or large DC polarization voltages^21^. More importantly, the in-air operation of piezoelectric devices at moderate *Q*-values allows for the implementation of mode-matched gyroscopes with a resonant bandwidth exceeding a few hundred hertz. However, contrary to capacitive devices that exploit the electrostatic softening mechanism for mode matching, piezoelectric gyroscopes require alternative techniques for frequency matching and modal alignment that can be applied independently of electrostatic transduction.

Dynamic frequency matching of piezoelectric gyroscopes has been demonstrated based on the application of electromechanical displacement feedback to adjust the effective stiffness of the drive mode without the need for electrostatic tuning^22–25^. Eigenmode operation has been introduced recently as a quadrature error cancellation technique for resonant piezoelectric gyroscopes based on the virtual rotation of the forcing and readout electrodes regardless of the transduction mechanism^26^. Concurrent application of these two techniques allows for perfect mode matching of resonant gyroscopes without the need for conventional electrostatic tuning or physical trimming methods^27–29^.

In this study, the theory of eigenmode operation based on modal analysis of an imperfect gyroscope is presented. The natural frequencies and natural mode shapes are derived assuming stiffness mismatch and cross-coupling between the two vibration modes. It is shown that by applying a linear transformation to the equations of motion, it is possible to decouple the two modes of the device to remove zero-rate error independently of the transduction mechanism. The eigenmode operation technique is implemented to align the vibration modes of a piezoelectric-on-silicon annulus resonant gyroscope. Complete mode matching of the device is shown by applying a displacement feedback signal to the drive mode, which in effect tunes the stiffness of the resonator for frequency matching. The performance of the mode-matched piezoelectric gyroscope is characterized, showing a low noise level while offering a much larger bandwidth than those of mode-matched capacitive devices.

## Results and discussion

### Theory of eigenmode operation

Coriolis vibratory gyroscopes are often modeled with a second-order system of differential equations, as shown below,

1 $$\left[ M \right]\left\{ {\ddot q} \right\} + \left[ C \right]\left\{ {\dot q} \right\} + \left[ K \right]\left\{ q \right\} = \left\{ Q \right\}$$

where $\left[ M \right]$, $\left[ C \right]$, and $\left[ K \right]$ are the mass, damping, and stiffness matrices defining the gyroscopic system, $\left\{ q \right\}$ is the generalized coordinate vector, and $\left\{ Q \right\}$ is the forcing vector. In an ideal vibratory gyroscope, energy is coupled between the two resonant modes only due to an applied external rotation. However, in practice, because of misalignment of the vibration direction with respect to the location of the input and output electrodes, energy is transferred between the two modes even in the absence of an external rotation signal. This coupling effect is modeled in the equations of motion of the gyroscope by introducing off-diagonal terms in the stiffness and damping matrices to cross-couple force to displacement and velocity between the two modes. The mismatch between the resonant frequencies of the two vibration modes is also modeled by the introduction of a stiffness mismatch term as given below

2 $$\left[ {\begin{array}{*{20}{c}} m & 0 \\ 0 & m \end{array}} \right]\left\{ {\begin{array}{*{20}{c}} {\ddot q_1} \\ {\ddot q_2} \end{array}} \right\} + \left[ {\begin{array}{*{20}{c}} {c - {\Delta}c} & {c_{12} - 2m{\Omega}} \\ {c_{12} + 2m{\Omega}} & {c + {\Delta}c} \end{array}} \right]\left\{ {\begin{array}{*{20}{c}} {\dot q_1} \\ {\dot q_2} \end{array}} \right\} + \left[ {\begin{array}{*{20}{c}} {k - {\Delta}k} & {k_{12}} \\ {k_{12}} & {k + {\Delta}k} \end{array}} \right]\left\{ {\begin{array}{*{20}{c}} {q_1} \\ {q_2} \end{array}} \right\} = \left\{ {\begin{array}{*{20}{c}} {Q_1} \\ {Q_2} \end{array}} \right\}$$

where $q_1$, $q_2$, $Q_1$, and $Q_2$ are the generalized coordinate and force components defined in the physical direction of gyroscope excitation and readout; $m$, $c$, and $k$ are the mass, damping, and stiffness coefficients for both modes; ${\Delta}c$ and ${\Delta}k$ represent the damping and stiffness mismatches between the two modes; $c_{12}$ and $k_{12}$ represent the damping and stiffness cross-coupling between the two modes; and ${\Omega}$ is the magnitude of the angular velocity vector, which is perpendicular to the plane of generalized coordinates.

### Modal analysis of the gyroscopic system

First, let us consider the case without mechanical damping where in the absence of an external rotation signal, the damping matrix reduces to zero. Considering zero forcing, we arrive at the homogeneous Eq. (3), which provides insight into the natural behavior of the system.

3 $$\left[ M \right]\left\{ {\ddot q} \right\} + \left[ K \right]\left\{ q \right\} = \left\{ 0 \right\}$$

The solution to the above homogeneous equation is of the form $\left\{ q \right\} = {\Re} \left( {B\left\{ \phi \right\}e^{j\omega t}} \right)$. Substituting this form into Eq. (3), we arrive at the following eigenvalue problem.

4 $$\left( {\left[ K \right] - \omega ^2\left[ M \right]} \right)\left\{ \phi \right\} = \left\{ 0 \right\}$$

Solving Eq. (4) results in the eigenfrequencies and eigenmodes of the system.

5 $$\omega _1 = \sqrt {\frac{{k - \sqrt {{\Delta}k^2 + k_{12}^2} }}{m}} ,\omega _2 = \sqrt {\frac{{k + \sqrt {{\Delta}k^2 + k_{12}^2} }}{m}}$$

6 $$\left\{ {\phi _1} \right\} = \left\{ {\begin{array}{*{20}{c}} 1 \\ {\frac{{{\Delta}k - \sqrt {{\Delta}k^2 + k_{12}^2} }}{{k_{12}}}} \end{array}} \right\},\left\{ {\phi _2} \right\} = \left\{ {\begin{array}{*{20}{c}} 1 \\ {\frac{{{\Delta}k + \sqrt {{\Delta}k^2 + k_{12}^2} }}{{k_{12}}}} \end{array}} \right\}$$

Equations (5) and (6) give the natural frequencies and natural directions of the vibration for a vibratory gyroscope with stiffness mismatch and cross-coupling between the two modes. It is important to note that even in the absence of any stiffness mismatch, the two vibration modes will still have different resonance frequencies due to stiffness cross-coupling between the two modes. It is also informative to consider the eigenvector directions for the two extreme cases where only the stiffness mismatch is set to zero or where only the stiffness cross-coupling is set to zero. The former case results in eigenmodes that are in the direction of the generalized coordinates or, in other words, in the physical direction of transduction electrodes, whereas the latter case results in eigenmodes that are 45° away from the direction of the transduction electrodes, which corresponds to equal direct and indirect coupling between the two modes.

To take advantage of the orthogonality of the eigenmodes, the modal masses $\mu _1$ and $\mu _2$ can be defined as given below^30^.

7 $$\mu _1 = \left\{ {\phi _1} \right\}^T\left[ M \right]\left\{ {\phi _1} \right\},\mu _2 = \left\{ {\phi _2} \right\}^T\left[ M \right]\left\{ {\phi _2} \right\}$$

The eigenvectors can be normalized by the modal masses to define the normal modes $\left\{ {{\Phi}_1} \right\}$ and $\left\{ {{\Phi}_2} \right\}$ and the normal mode matrix $\left[ {\Phi} \right]$ as follows.

8 $$\left\{ {{\Phi}_1} \right\} = \frac{1}{{\sqrt {\mu _1} }}\left\{ {\phi _1} \right\},\left\{ {{\Phi}_2} \right\} = \frac{1}{{\sqrt {\mu _2} }}\left\{ {\phi _2} \right\}$$

9 $$\left[ {\Phi} \right] = \left[ {\left\{ {{\Phi}_1} \right\}\left\{ {{\Phi}_2} \right\}} \right]$$

The normal modes are orthogonal with respect to the mass and stiffness matrices, as shown in Eqs. (10) and (11),

10 $$\left[ {\Phi} \right]^T\left[ M \right]\left[ {\Phi} \right] = \left[ I \right]$$

11 $$\left[ {\Phi} \right]^T\left[ K \right]\left[ {\Phi} \right] = \left[ {\omega _{nat}^2} \right]$$

where $\left[ I \right]$ is the identity matrix and $\left[ {\omega _{nat}^2} \right]$ is a diagonal matrix consisting of the square of the eigenfrequencies. Based on the normal mode matrix, the following linear transformation between the generalized coordinates $\left\{ q \right\}$ and the modal coordinates $\left\{ \eta \right\}$ can be considered.

12 $$\left\{ q \right\} = \left[ {\Phi} \right]\left\{ \eta \right\}$$

Substituting for $\left\{ q \right\}$ in the differential equations of motion of the gyroscope, we obtain

13 $$\left[ M \right]\left[ {\Phi} \right]\left\{ {\ddot \eta } \right\} + \left[ K \right]\left[ {\Phi} \right]\left\{ \eta \right\} = \left\{ Q \right\}$$

Left-multiplying Eq. (13) with the transpose of the normal mode matrix, $\left[ {\Phi} \right]^T$, and applying the orthogonality properties in Eqs. (10) and (11) result in the diagonalization of the mass and stiffness matrices. In other words, the modal cross-coupling is removed under normal modal transformation.

14 $$\left\{ {\ddot \eta } \right\} + \left[ {\omega _{nat}^2} \right]\left\{ \eta \right\} = \left[ {\Phi} \right]^T\left\{ Q \right\}$$

### Eigenmode operation

**Fig. 1:**
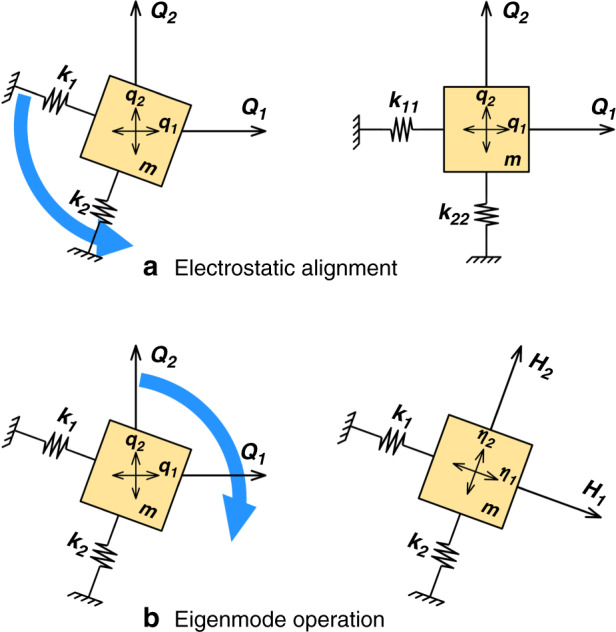
Schematic representation of modal alignment techniques. **a** The electrostatic alignment technique corrects the misalignment in the stiffness direction to remove quadrature coupling. **b** Eigenmode operation adjusts the excitation and readout directions to the as-fabricated natural vibration direction

The reason for the appearance of zero-rate error, or more specifically quadrature error, in the response of resonant gyroscopes is the existence of off-diagonal terms in the system of equations of motion that cause coupling of the two vibration modes. This misalignment of the mode shape with respect to the transduction direction is commonly corrected by aligning the direction of vibration to the transduction direction via application of electrostatic forces, as shown in Fig. 1a.

Equation (14) represents a new set of modal coordinates, $\left\{ \eta \right\}$, along which there is no cross-coupling between the two modes of the gyroscope. Therefore, it is alternatively possible to remove the zero-rate error by aligning the transduction direction to the eigenmode direction independently of the transduction mechanism. The method of operating the gyroscope in the direction of the natural vibration modes of the structure is herein referred to as eigenmode operation, as depicted in Fig. 1b.

The transformation from $\left\{ q \right\}$ to $\left\{ \eta \right\}$ is via the transpose of the modal matrix, $\left[ {\Phi} \right]^T$ which, in essence, represents a rotation transformation for the gyroscopic system. In other words, there exists a direction in the plane of the original generalized coordinates along which the two modes of the gyroscope are uncoupled. To be strictly accurate, it is worth noting that $\sqrt m \left[ {\Phi} \right]^T$ is the pure rotation matrix, but the presence of a scaling factor is not crucial to our discussion. The desired forcing vector for uncoupled operation is $\left\{ {\rm{H}} \right\} = \left[ {\Phi} \right]^T\left\{ Q \right\}$, and the desired readout vector is $\left\{ \eta \right\} = \left[ {\Phi} \right]^T\left\{ q \right\}$. Therefore, both the excitation and readout directions must be transformed by the $\left[ {\Phi} \right]^T$ matrix to ensure decoupling.

To implement the transformation, we can use pairs of electrodes that span the two-dimensional subspace of the plane normal to the axis of rotation for gyroscope excitation and readout. For instance, by applying the scaling coefficients of $\alpha$ and $\left( {1 - \alpha } \right)$ to each pair, it is possible to sweep the effective direction of transduction between the two electrodes, in effect creating a set of virtual electrodes that are aligned to the direction of eigenmodes.

Theoretically, the scaling coefficient $\alpha$ is equal at the excitation and readout transducers of the gyroscope. However, to compensate for mismatch effects, the input and output scaling coefficients can be set slightly differently. In addition, the scaling coefficients applied to the electrodes should be normalized depending on the angle between the electrodes to keep the total magnitude of excitation and readout constant as we sweep the direction of transduction. Assuming an angle of $\theta$ between the two members of each electrode pair, the scaling coefficients of $\alpha$ and $\left( {1 - \alpha } \right)$ need to be normalized by $\sqrt {2\alpha \left( {\alpha - 1} \right)\left( {1 - \cos \theta } \right) + 1}$ to keep the transduction magnitude constant.

### The effect of damping on eigenmode operation

If the damping matrix is proportional to the mass and stiffness matrices, $\left[ C \right] = \beta \left[ M \right] + \gamma \left[ K \right]$, the same transformation using the normal mode matrix results in the diagonalization of the damping matrix and the stiffness matrix^30^, which means that the equations of motion will still be uncoupled, and eigenmode operation using the same scaling coefficients removes the zero-rate output, as derived below.

15 $$\left[ {\Phi} \right]^T\left[ M \right]\left[ {\Phi} \right]\left\{ {\ddot \eta } \right\} + \left[ {\Phi} \right]^T\left( {\beta \left[ M \right] + \gamma \left[ K \right]} \right)\left[ {\Phi} \right]\left\{ {\dot \eta } \right\} + \left[ {\Phi} \right]^T\left[ K \right]\left[ {\Phi} \right]\left\{ \eta \right\} = \left[ {\Phi} \right]^T\left\{ Q \right\}$$

16 $$\left\{ {\ddot \eta } \right\} + \left( {\beta + \gamma \left[ {\omega _{nat}^2} \right]} \right)\left\{ {\dot \eta } \right\} + \left[ {\omega _{nat}^2} \right]\left\{ \eta \right\} = \left[ {\Phi} \right]^T\left\{ Q \right\}$$

Because of the axial symmetry of degenerate resonant gyroscopes, it is acceptable to assume that even if the damping matrix is not exactly proportional to the mass and stiffness matrices, it is almost proportional to those matrices. It should be noted that the coupling caused by cross-damping is always 90° apart from that caused by the off-diagonal stiffness terms. Off-diagonal damping terms result in a residual coupled signal that is in phase with the output due to external rotation under mode-matched conditions. Therefore, compensation for this term is not possible without affecting the response of the gyroscope to the rate signal. Measurement results show that the total magnitude of the zero-rate output decreases as the effective direction of transduction approaches the eigenmode direction, which suggests that the damping matrix is either almost proportional to the mass and stiffness matrices or that the off-diagonal terms of the damping matrix are very small.

### AlN-on-Si annulus resonant gyroscope

It has been previously demonstrated that in contrast to capacitive BAW gyroscopes, which commonly use elliptical vibration mode shapes, the degenerate in-plane flexural vibration mode shapes of plate structures should be used to provide strong electromechanical coupling with thin-film piezoelectric transduction for the realization of mode-matched resonant gyroscopes. Different axisymmetric structures can support the in-plane flexural mode shape, and this work presents the implementation of a piezoelectric gyroscope using an annulus structure with a network of supporting tethers that is designed to acoustically decouple the resonant device from the surrounding substrate.

**Fig. 2:**
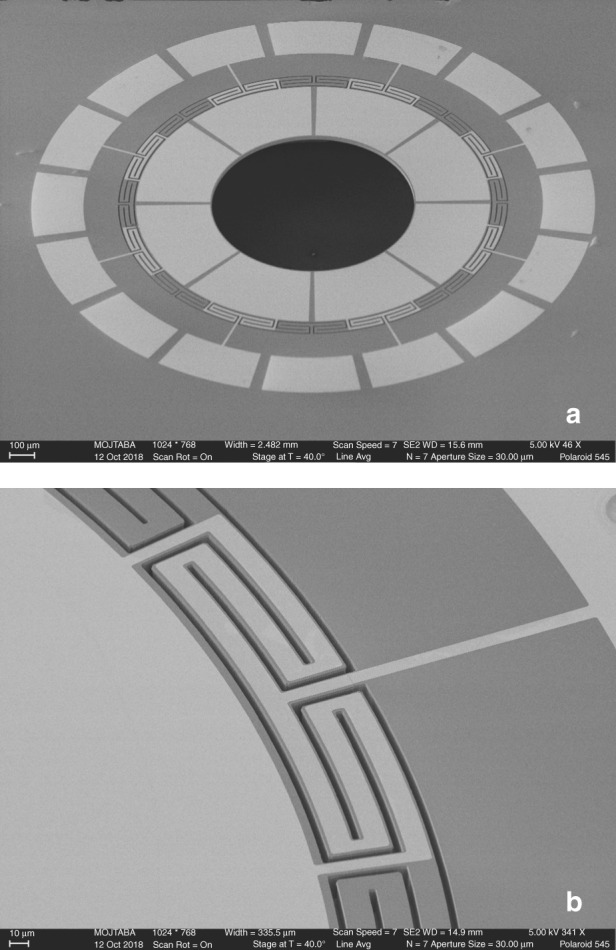
SEM images of the annulus piezoelectric resonant gyroscope fabricated using a simple four-mask AlN-on-Si process. **a** Eight top electrodes used for eigenmode operation of the gyroscope. **b** The network of peripheral tethers is used to both support the structure and provide a routing path for the electrical connections

A scanning electron microscope (SEM) image of the annulus piezoelectric gyroscope is shown in Fig. 2a. The device structure comprises a 1.3 µm thin film of aluminum nitride (AlN) deposited between 50 nm top and bottom molybdenum (Mo) electrode layers and stacked on top of a 15 µm-thick (100) silicon device layer. The silicon handle layer is etched from the back side to release the structural device layer.

The annulus structure has an outer radius of 700 µm, an inner radius of 400 µm, a radial tether span of 0.8°, and a tangential tether width of 15 µm. The radial trench span is 0.4° and the tangential trench width etched into the silicon device layer is 5 µm. The device is anchored to the substrate through a network of circularly symmetric double-T supports to ensure structural symmetry of the drive and sense modes. The tether and trench widths are designed for maximum acoustic decoupling of the double-T structure. The support network is also used to route the electrical signals to the eight metal electrodes attached to the structure. Figure 2b shows a close-up image of one section of the supporting tethers with the routing of the top Mo layer between the device and a signal pad. The signal electrode pads on the substrate are separated by eight ground electrodes, which provide a common connection to the bottom Mo layer around the device.

The gyroscope is transduced using eight identical top electrodes. Pairs of electrodes that are spatially 45° apart are used for gyroscope excitation and readout compatible with eigenmode operation. One pair is used to excite the drive mode, a second pair is used to pick off the drive signal, and the remaining two pairs of electrodes are used for differential readout of the sense-mode signal.

**Fig. 3:**
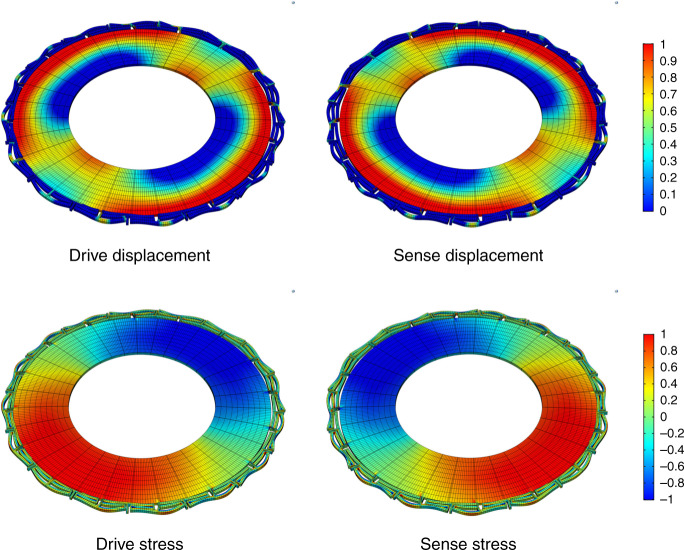
Drive and sense vibration mode shapes of the annulus piezoelectric gyroscope showing the displacement field (top) and the stress pattern (bottom) of the in-plane flexural resonant mode. The stress field pattern is suitable for top-side piezoelectric transduction

The displacement and stress field patterns for the pair of degenerate first-order in-plane flexural gyroscopic vibration modes are shown in Fig. 3. The orthogonality of the two mode shapes provides a means for detection of the Coriolis-induced signal for measurement of the angular rate of rotation and the uniform stress field pattern of the flexural mode shape ensures efficient piezoelectric transduction using the top thin film piezoelectric material, resulting in a low motional resistance, a large-scale factor, and small mechanical equivalent rotation noise for the resonator gyroscope.

### Experimental results

Eigenmode operation circuitry for the piezoelectric annulus gyroscope was implemented on a printed circuit board and the device was interfaced digitally with a field-programmable gate array (FPGA) module from National Instruments (NI) to realize the drive loop, the sense channel, and the dynamic frequency tuning scheme. The two excitation signals of the gyroscope are scaled using two voltage dividers followed by buffers to apply the coefficients of $\alpha$ and $\left( {1 - \alpha } \right)$ with the proper normalization factor to the drive electrodes. The same scaling coefficients are used to scale the drive output and the differential sense output signals to generate the effective drive and sense currents from the pick off electrodes. The effective drive current is integrated and scaled in a digital feedback loop to provide the displacement signal necessary for dynamic frequency tuning. The level of frequency tuning is adjusted by an external coefficient to control the amplitude of the displacement feedback.

Perfect mode matching, where the sense-mode response is minimized and the drive and sense frequencies are equal, is achieved by iterative adjustment of the factor $\alpha$ and matching the drive and sense frequencies by adjusting the level of displacement feedback.

**Fig. 4:**
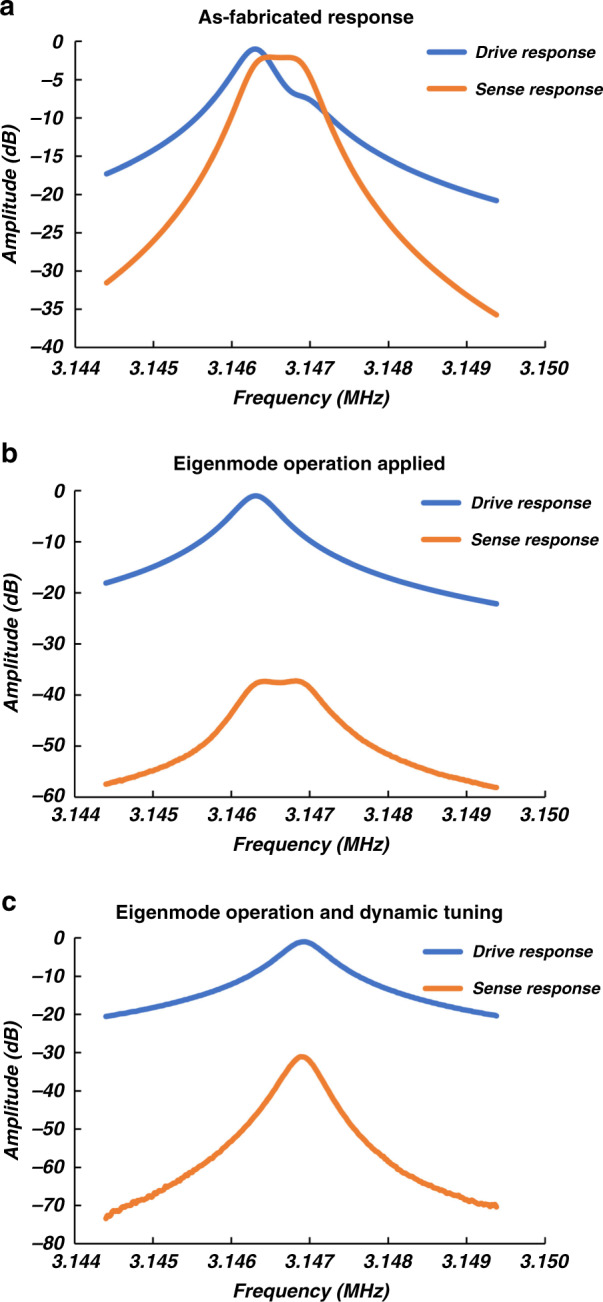
Frequency response of the AlN-on-Si resonant gyroscope. The normalized drive and sense frequency responses of the AlN-on-Si resonant gyroscope as fabricated (**a**), with eigenmode operation only (**b**), and with eigenmode operation and dynamic frequency tuning applied (**c**). The zero-rate error is reduced by ~35 dB with the application of eigenmode operation. The device shows a *Q* of ~6300 while operating in air, corresponding to a mode-matched mechanical bandwidth of 500 Hz

The normalized drive and sense frequency responses of the AlN-on-Si resonant gyroscope as fabricated, with eigenmode operation only, and with eigenmode operation and dynamic frequency tuning applied simultaneously are shown in Fig. 4. The amplitudes are normalized with respect to the drive amplitude at resonance, which was measured to be approximately −23 dB, translating to a motional resistance of ~1300 Ω. The zero-rate error is suppressed most when the direction of the virtual transduction electrode is closest to the eigenmode direction, causing the sense-mode peak to disappear from the drive frequency response and the sense-mode response to be reduced significantly. The zero-rate error is reduced by ~35 dB with the application of eigenmode operation. The device shows a *Q* of ~6300 while operating in air, corresponding to a large mode-matched mechanical bandwidth of ~500 Hz.

**Fig. 5:**
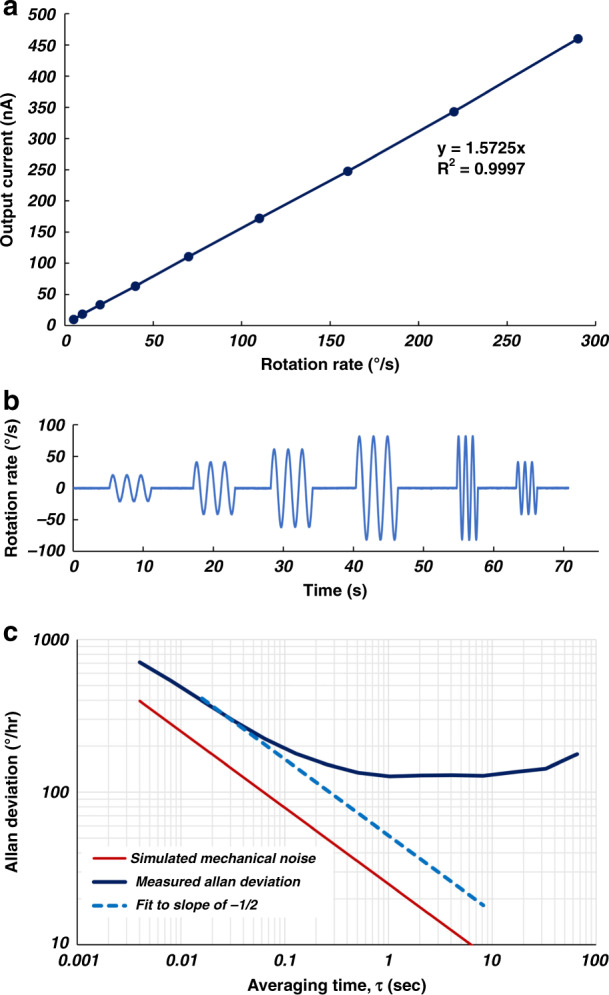
Rotation rate and bias response of the AlN-on-Si resonant gyroscope. **a** The measured output current of the piezoelectric gyroscope vs. the applied external rotation rate. The experimental scale factor is 1.57 nA/°/s for an input 90 mV peak drive voltage. **b** The time-domain output waveform for different rotation rates at two frequencies of 1 and 2 Hz, and different rotation amplitudes. **c** Allan deviation plot of the piezoelectric gyroscope showing a measured angle random walk of 0.86°/√h, which is close to the simulated MNEΩ of 24.7°/h/√Hz considering the effect of interface noise. The bias instability of the gyroscopes is ~125°/h, which is limited by the precision and excess noise of the interface circuitry

The measured output current of the piezoelectric gyroscope as a function of the applied external rotation rate is plotted in Fig. 5a. The experimental scale factor is measured to be 1.57 nA/°/s for a 90 mV-peak input drive voltage. The time-domain output waveform of the gyroscope is plotted in Fig. 5b over a period of 70 s. The input mechanical rotation rate to the device is 20, 40, 60, 80, 40, and 20°/s, applied at a frequency of 1 Hz for the first four signals and 2 Hz for the last two. The Allan deviation plot of the piezoelectric gyroscope showing a measured angle random walk of 0.86°/√h is given in Fig. 5c. The measured thermal noise value is very close to the simulated mechanical noise equivalent rotation rate (MNEΩ) of 24.7°/h/√Hz considering the effect of the interface noise. The bias instability of the gyroscope is ~125°/h, which is limited by the precision of modal alignment and excess noise from the discrete electronics. The precision of modal alignment is limited by the resolution of the eigenmode coefficient adjustment. The analog potentiometers that are used at the input and output ports of the gyroscope to adjust the coefficients of eigenmode operation impose a significant limitation on the resolution of the eigenmode coefficients and hence limit the level of decoupling between the drive and sense modes, which directly affects the bias instability of the device. The other limiting factor in achieving a lower bias instability is the phase noise of the drive signal, which is generated by the numerically controlled oscillator (NCO) and digital-to-analog converters^31^. The phase noise appears as a signal-dependent flicker component at the output of the gyroscope and hence limits the minimum achievable bias instability regardless of the level of the excitation signal. Further characterization data are provided in the Supplementary Information.

**Fig. 6:**
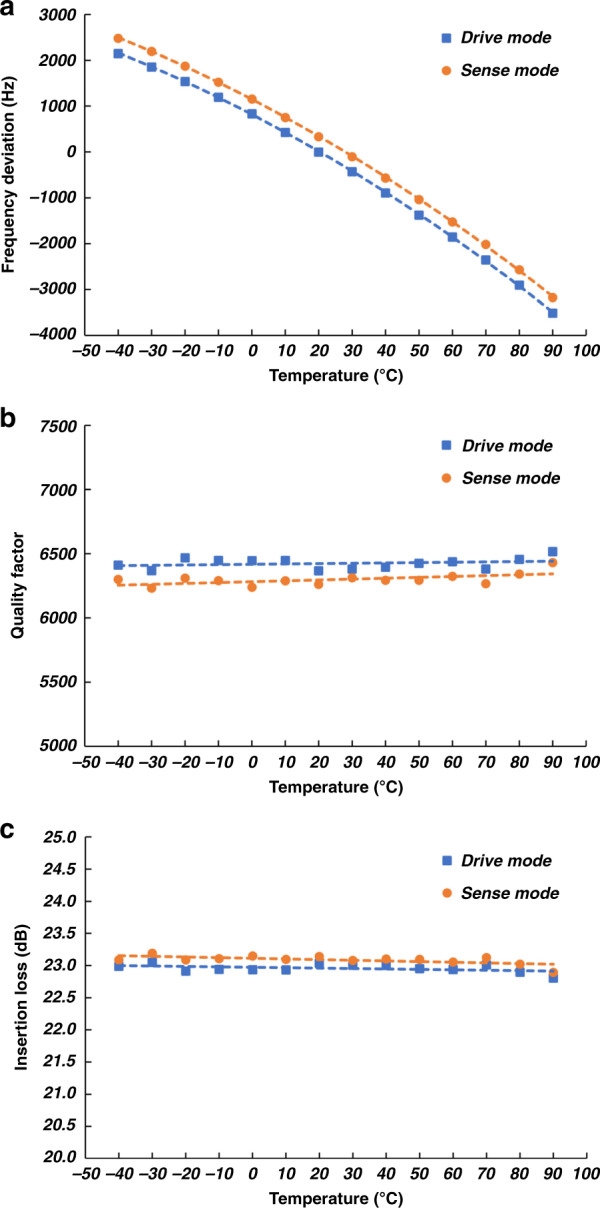
Temperature characterization plots for the AlN-on-Si resonant gyroscope. **a** Temperature dependence of drive and sense resonance frequency deviations from the drive resonance frequency at 20 °C showing a maximum frequency split of 107 p.p.m between the two modes with a maximum frequency split variation of 6 p.p.m. over the temperature range. The linear temperature coefficient of frequency is approximately −12 p.p.m./°C. **b** Drive and sense-mode insertion loss vs. temperature. The insertion loss of both modes remains almost constant over the temperature range with a maximum difference of 0.9% between the two modes and maximum variation of 0.6%. **c** Drive and sense-mode *Q* vs. temperature. Considering mechanical decoupling from the substrate, the quality factor remains almost constant over the temperature range with a maximum difference of 3.2% between the two modes and a maximum variation of 2.2%

The temperature behavior of the drive and sense resonance modes of the piezoelectric gyroscope is characterized from −40 °C to +100 °C, as shown in Fig. 6. The maximum frequency split between the two modes is 107 p.p.m. with a maximum frequency split variation of 6 p.p.m. over the temperature range. The linear temperature coefficient of frequency is approximately −12 p.p.m./°C. The insertion losses of the drive and sense modes remain almost constant over the temperature range, with a maximum difference of 0.9% between the two modes and a maximum variation of 0.6% over the temperature range. Considering mechanical decoupling from the substrate and homogeneous piezoelectric losses, the quality factors of the two modes remain almost constant over the temperature range with a maximum difference of 3.2% between the two modes and a maximum variation of 2.2% from −40 °C to +100 °C.

## Materials and methods

### Finite element analysis

The performance of the piezoelectric annulus gyroscope was simulated using the COMSOL finite element analysis tool. The design was first optimized to minimize anchor loss and suppress any spurious modes close to the frequency of operation. Anchor loss was computed using perfectly matched layers, and the anchor quality factor (*Q*) value obtained from simulations was larger than 2,000,000, ensuring structural decoupling from the substrate. For simulation of the scale factor and MNEΩ of the device, a *Q*-value of 6300, which was obtained from measurement of the devices after fabrication, and a driving voltage of 90 mV, according to the testing conditions, were assumed. The simulated Coriolis output current results in a scale factor of 1.62 nA/°/s. The simulated motional resistance is 1082 Ω, and the maximum kinetic energy of the drive mode is computed to be 0.593 nJ, corresponding to a gyroscopic coupling coefficient of 0.876 and MNEΩ of 24.7°/h/√Hz. The small motional resistance value proves the strong electromechanical coupling of the thin-film piezoelectric layer for transduction of the in-plane flexural vibration modes.

### Fabrication process

**Fig. 7:**
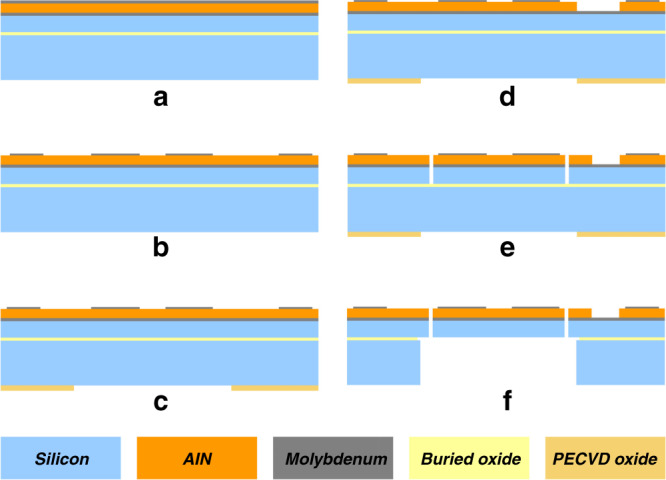
Fabrication process flow for the AlN-on-Si resonant gyroscope. **a** Deposit the piezoelectric stack on an SOI wafer, **b** pattern the top Mo electrode layer, **c** deposit and pattern the backside oxide mask, **d** pattern the AlN layer to access the bottom Mo electrode, **e** etch the silicon device layer, and **f** etch the handle layer and release the device

A cross-sectional view of the four-mask fabrication process for the piezoelectric gyroscope is depicted in Fig. 7. The process begins with the deposition of the piezoelectric stack, which consists of a thin film of sputtered AlN sandwiched between a top signal Mo layer and a bottom ground Mo layer, on a silicon-on-insulator substrate. Next, the top Mo electrode layer is patterned to define the signal electrodes and pads. Then, an oxide mask is deposited on the backside of the wafer and patterned for subsequent handle-layer etching. After that, the AlN layer is patterned to open access paths to the bottom Mo layer for ground connections. Finally, the silicon device layer is etched to define the device structure, the silicon handle layer is etched for backside release of the structure, and the device is released with a short step of hydrofluoric acid oxide etching.

### Digital interface architecture

**Fig. 8:**
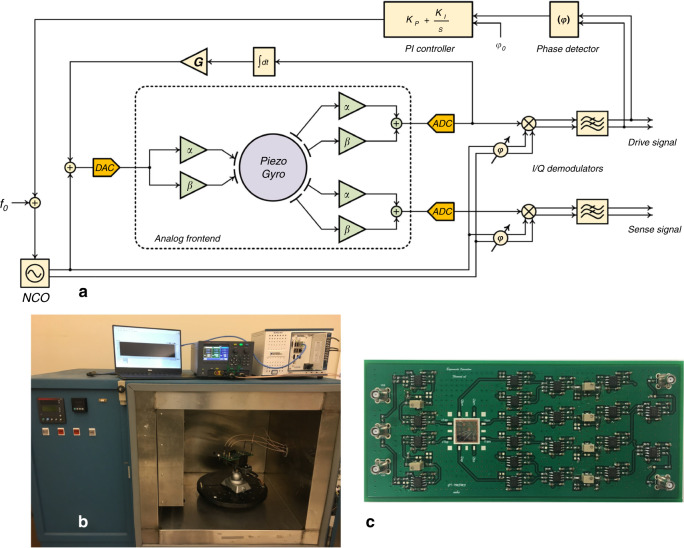
Interface architecture and measurement setup for the piezoelectric gyroscope. **a** Interface system architecture of the piezoelectric gyroscope. The drive loop, sense channel, and dynamic displacement feedback are implemented in the FPGA in the digital domain. The piezoelectric gyroscope is actuated and sensed in the analog domain based on eigenmode operation. **b** Measurement setup, including a FlexRIO system from National Instruments in communication with the analog frontend mounted on the rate table and controlled by the PC. **c** Printed circuit board showing the device actuated by applying the eigenmode input pair voltage and sensed by processing the output current according to eigenmode operation. Potentiometers at the input and output are used for realization of the eigenmode operation coefficients

The digital interface was implemented on a FlexRIO system from NI, which consists of an FPGA in communication with a real-time controller^32,33^. An adapter module from NI provides analog-to-digital and digital-to-analog conversion capability. A schematic of the interface system for the piezoelectric gyroscope is shown in Fig. 8. The drive oscillator loop, the sense channel and the electromechanical feedback loop for the realization of frequency tuning are all implemented in the digital domain. The drive input signal is generated using an NCO. The frequency of the NCO is controlled based on the output phase of the drive demodulated signal to track the resonance peak using a proportional-integral controller. The sense channel demodulators use the same NCO-generated signal for demodulation of the gyroscope output signal with a phase shift applied to account for the delay between the input and output signals to the gyroscope. The frequency tuning loop is implemented using the output drive signal. The output drive signal is proportional to the velocity of the resonator. Therefore, this signal is integrated and applied back at the input with an amplification gain to control the displacement-dependent signal at the input of the gyroscope. Implementing the eigenmode operation circuitry in the digital domain results in a large flicker noise in the output signal of the gyroscope mainly due to the large uncorrelated noise injected in the system by using two different digital-to-analog converters. For that reason, eigenmode operation processing is performed in the analog domain. The drawback is the limited precision of implementation of the eigenmode coefficients, which directly affects the output bias of the gyroscope.

## Conclusions

In this study, we presented the theory of eigenmode operation of Coriolis vibratory gyroscopes and its implementation on a thin-film AlN-on-Si gyroscope. The sputter-deposited thin-film piezoelectric material used in our device offers significant advantages in terms of the manufacturability of MEMS gyroscopes compared with the current dominant technology based on bulk piezoelectric materials. The utilization of the degenerate pair of in-plane flexural vibration modes of an annular structure in this work is the key feature that enables efficient thin-film piezoelectric transduction, resulting in low noise, a low motional resistance, and a high-scale factor suitable for low-power applications. To provide mode matching capability, we showed analytically that modal alignment of resonant gyroscopes can be achieved by applying a rotation transformation to the actuation and sensing directions regardless of the transduction mechanism. We applied this eigenmode operation technique to mode match a piezoelectric gyroscope without the need for narrow capacitive gaps or DC polarization voltages, resulting in an ~35 dB reduction in modal coupling at the resonance frequency. We demonstrated a mode-matched operation bandwidth of ~250 Hz, along with a motional resistance of ~1300 Ω, a scale factor of 1.57 nA/°/s, and a measured angle random walk of 0.86°/√h. The gyroscope bias instability of 125°/h is mainly limited by the precision of the interface circuitry and excess noise from the discrete electronics. It is worth noting that the application of eigenmode operation is not limited to piezoelectric devices and can be extended to modal alignment of resonant gyroscopes regardless of their transduction mechanism or axis of operation. In particular, as electrostatic quadrature cancellation in pitch and roll gyroscopes requires the implementation of physical electrodes that span the subspaces of both in-plane and out-of-plane vibration modes to compensate for modal cross-coupling, eigenmode operation is a good candidate for modal alignment of those devices without the need for the implementation of complex fabrication processes.

## Supplementary information

Supplementary Information
