# Supplementary material for: Eigenmode operation of piezoelectric resonant gyroscopes
Source: Microsyst Nanoeng. 2020 Nov 30;6:108. doi: 10.1038/s41378-020-00204-3 (PMC8433452; doi:10.1038/s41378-020-00204-3)
Supplement: Supplementary file 1 — Supplementary Information [file 41378_2020_204_MOESM1_ESM.docx]

Supplementary Information for

Eigenmode Operation of Piezoelectric Resonant Gyroscopes

Mojtaba Hodjat-Shamami^1^ and Farrokh Ayazi^2^

^1,2^ School of Electrical and Computer Engineering, Georgia Institute of Technology, Atlanta, GA 30308, USA

Email: ^1^[shamami@gatech.edu](mailto:shamami@gatech.edu); ^2^[ayazi@gatech.edu](mailto:ayazi@gatech.edu)

# The effect of eigenmode operation on bias instability

The main factor affecting the bias instability (BI) of the gyroscope is the coupling between the drive and sense mode signals. By changing the coefficients of eigenmode operation at the input and output ports, the effective direction of transduction is adjusted to be aligned to the natural directions of vibration along which we get the maximum decoupling between the two modes and lowest bias level and bias instability. The maximum decoupling achievable depends on the precision of eigenmode operation adjustment and is limited by the analog potentiometers used in the interface circuitry. The Allan deviation plots are shown in Fig. S1 with and without eigenmode operation. The eigenmode operation resulted in significant reduction of bias instability from 1700°/hr to 270°/hr for a drive excitation voltage of 50mV-peak. The higher value of angle random walk (ARW) for the case where eigenmode operation is not applied is attributed to the larger frequency mismatch caused by the quadrature signal.


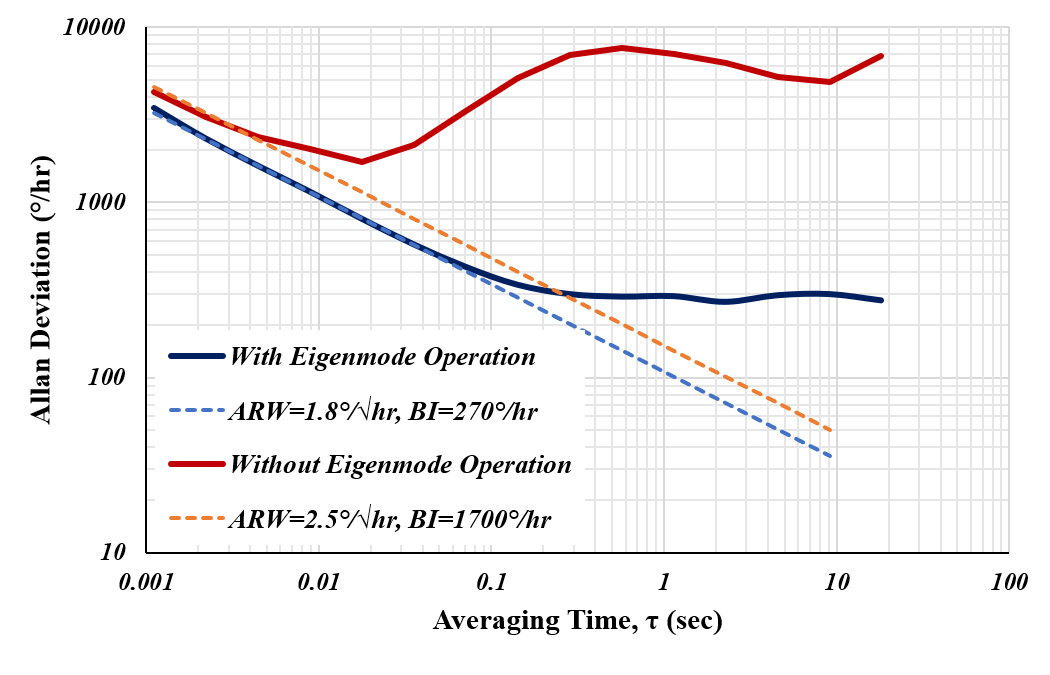


Fig. S1: Allan deviation plots with and without eigenmode operation. The bias instability is reduced from 1700 deg/hr to ~300 deg/hr by adjusting the eigenmode coefficients and hence the transduction direction. The residual bias is due to slight misalignment from the natural vibration directions caused by the limited resolution of eigenmode coefficients adjustment.

# The effect of phase noise on bias instability

The other limiting factor in achieving lower bias instability is the phase noise of the drive signal which is generated by the numerically controlled oscillator (NCO) and digital-to-analog converters (DACs) in our interface setup. The phase noise of the excitation signal appears as a flicker component in the bias output after demodulation and more importantly, the level of this flicker component is proportional to the level of the drive signal. This means that improving the scale factor by driving the gyroscope harder, does not translate into an improvement in the bias instability level as the flicker noise at the output increases proportionally. We have shown this effect by measuring the Allan deviation at two different drive voltage levels. As we can see below in Fig. S2, increasing the drive amplitude from 20mV-peak to 50mV-peak results in a proportional reduction in the ARW as the thermal noise remains constant. However, the BI remains almost constant at around 300 °/hr limited by the drive signal-proportional flicker component. (The BI level shown here is larger than what is reported in the main article as the decoupling between the drive and sense mode was different for these measurements.)


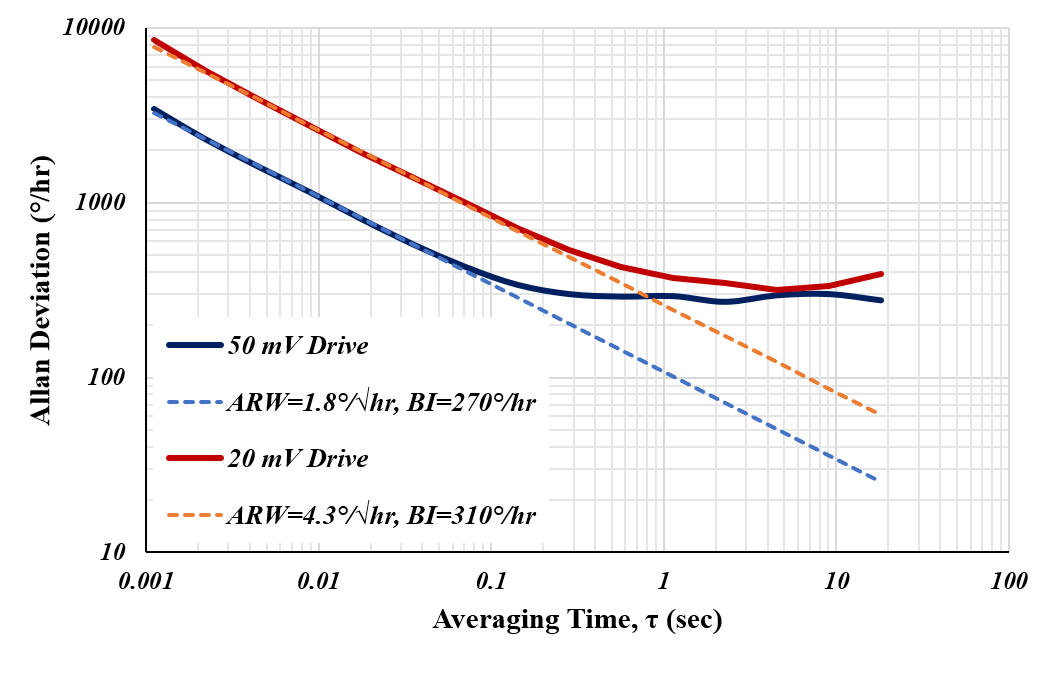


Fig. S2: Allan deviation plots at two different drive voltage levels. Increasing the excitation voltage from 20mV to 50mV results in the reduction of thermal noise in the output bias as expected. However, this reduction does not translate to lower bias instability as it is limited by the phase noise of the NCO and DACs (flicker noise after demodulation) which is proportional to the excitation signal. In other words, larger excitation results in larger scale factor and hence lower ARW as the thermal noise remains constant but it does not result in lower bias instability as the flicker noise is signal dependent.
